# Unlocking the potential of rural social enterprise

**DOI:** 10.1016/j.jrurstud.2017.12.021

**Published:** 2019-08

**Authors:** Artur Steiner, Simon Teasdale

**Affiliations:** Glasgow Caledonian University, UK

**Keywords:** Rural, Social enterprise, Community development, Economic development, Community cohesion

## Abstract

In this paper, we argue that social enterprise could represent a means of tackling rural challenges of providing sustainable economic development, addressing the withdrawal of public services and promoting community cohesion. The paper draws upon a review of existing academic as well as policy literature and develops a conceptual framework that helps to understand how to unlock the potential contribution of social enterprises to rural development. Drawing on an exploratory study conducted in two rural areas of Scotland we use interview data from social enterprise stakeholders to populate the conceptual framework and its rural (geographic), policy and social enterprise domains. Our study suggests that social enterprises can potentially enable an integrated approach to addressing local issues at the local level. They can create locally responsive services that fit the rural context. However, unlocking the potential of rural social enterprise may require moving beyond traditional policy silo approaches that treat economic development, community cohesion and public services as separate and disconnected since national policy-making frameworks have not always translated into practice at the rural level. Additionally, policy treatment of social enterprise needs to move beyond efforts to ‘scale up’ and achieve economies of scale. Collaborations between groups of social enterprises, and between social enterprises and public authorities can lead to economies of scope, particularly where strong trust-based relations within communities harness self-help and the co-production of services. With appropriate guidance and support, many rural challenges and needs could be transformed into opportunities for social enterprise development. In highlighting the opportunities and challenges faced by rural social enterprises, the paper suggests potential research gaps that, if filled, could contribute towards recognising and unlocking their full potential.

## Highlights

• Rural social enterprise conceptual framework combines rural, policy and social enterprise domains.
• Social enterprises recognise the interconnectedness between different rural needs and potential solutions.
• Rural challenges could be transformed into opportunities for social enterprise and community development.
• National social enterprise policies are not well-translated into practice at the rural level.
• Future success or rural social enterprises depends on breaking down traditional policy silo.

## 1 Introduction

Social enterprises enjoy growing political support around the globe. In the European Union, for example, social enterprises are supported through a number of initiatives that promote the creation and development of this form of organisation (European Commission, 2017). UK and Scottish policies are considered to be at the vanguard in terms of creating an environment that is supportive of social enterprise (Roy et al., 2015, Nicholls, 2010). This policy support has been associated with a wider neoliberal policy shift whereby the state is gradually withdrawing from its role in direct welfare provision, and public contracts are increasingly outsourced (Chaney and Wincott, 2014, Steinerowski et al., 2008). Simultaneously there has been increasing interest in the delivery of services by non-state players, and an acceptance that citizens’ involvement in the design and delivery of services should be facilitated (Milligan and Fyfe, 2005). Considerable resources have been devoted towards encouraging third sector organisations to become more socially enterprising, whether through grants to help them achieve financial sustainability, business support, or the outsourcing of public services (Nicholls and Teasdale, 2016).

Successive UK governments from New Labour (1997–2010), the Conservative-led coalition government (2010–15) and the Conservative government (2015-) have emphasised the importance of social enterprise in the delivery of public services and their contribution towards community cohesion. The Conservative manifesto prior to the 2015 general election set out a vision for ‘a more engaged nation, one in which we take more responsibility for ourselves and our neighbours; communities working together’ (Conservative Party, 2015:45). The extent to which this rhetoric has been matched (at a UK level) by policy action is debatable, with some suggesting that this vision has been little more than a smokescreen for the dismantling of the welfare state (Taylor-Gooby and Stoker, 2011). However, just as social enterprise covers a wide range of organisational forms, it is possible to see different versions of neoliberalism operating across different contexts (Nicholls and Teasdale, 2016). Scotland, while part of the United Kingdom, has devolved responsibility in many policy areas, including (for the most part) social enterprise (Alcock, 2012). Some commentators have speculated that the commitment to community cohesion and social enterprise is greater in Scotland than the rest of the UK, whether in part because of a historical commitment to collectivist approaches, or simply as a rejection of a Westminster style approach to politics (Roy et al., 2015). From this perspective, social enterprise in Scotland, at least in theory, could be said to operate as a more equal partner in the co-design and delivery of services rather than as an agent of the state.

Despite increasing interest within policy, practice and academia in relation to the (potential) role of social enterprises as service providers (Teasdale, 2012a), and in spite of growing recognition that geographical context matters, relatively little is known about the activities of *rural* social enterprises (Munoz et al., 2015). Although it would appear that social enterprises are relatively prevalent in rural areas, their contribution to rural development and solving rural challenges is neither fully recognised by academics nor utilised by policymakers. In this paper we begin to address this knowledge gap. First we draw on existing academic and policy literature in order to develop an initial conceptual framework that helps to understand how to unlock the potential contribution of social enterprises to rural development. Subsequently we draw on interview data from an exploratory study conducted in rural Scotland to begin to ‘populate’ this framework. Incorporating perceptions of social enterprise stakeholders on how the potential for rural social enterprise might be better realised helps to better understand the potential impact of social enterprises on rural development, the opportunities and challenges they face, and research gaps which, if filled, could help catalyse this embryonic research field.

## 2 Rurality, social enterprises and the policy background

### 2.1 Rural domain

Rural areas frequently face challenges of limited economic development due to low profitability, the withdrawal of public services seen as economically unviable, and challenges to community cohesion caused by demographic and geographical factors. Economically, rurality is frequently associated with the inaccessibility of goods and services (Smailes, 2002). This specific geographical context affects the activities of businesses and process of entrepreneurship (Korsgaard et al., 2015). A sparsely populated landmass not only presents challenges for people in accessing goods and services but also creates problems for businesses in connecting with widely dispersed clients and recruiting skilled employees who can help to grow a business (Steiner and Cleary, 2014). Physical and technical barriers, and distance from main service and support centres (Schouten et al., 2012) also represent obstacles for business development (Boshworth, 2012). This detracts from the ability of businesses to grow and achieve economies of scale and, frequently, impacts on their economic sustainability. Narrow economic development opportunities discourage mainstream commercial businesses from investing their financial resources in rural locations, leaving market gaps as well as limited employment opportunities for those living in rural areas (Galloway, 2007).

Due to high costs per capita, rural areas may also suffer additional social challenges compared to urban centres, particularly in a context of public spending cuts, and they have been disproportionately affected by an increasing withdrawal of physical public services (Hodge et al., 2017). For instance, healthcare organisations have moved to larger regional centres leaving smaller rural towns and villages with limited (or no) primary healthcare services (Farmer and Nimegeer, 2014). In many rural places, local schools, libraries, transport and postal as well as social and emergency services have been withdrawn as a part of a wider movement towards, ironically, more ‘efficient’ economies. Consequently we see increasingly persistent pockets of rural deprivation (Steiner and Atterton, 2015).

Rural communities in sparsely populated areas experience limited opportunities for wider social interaction. They also face out-migration of young people and in-migration of retirees, leading to an ageing rural population (Farmer et al., 2011). A lack of local services such as local pubs, shops and post offices means that there are less opportunities for social interaction, and the strong ties once associated with rural communities are being eroded. The character of many agricultural or fishing villages, and rural market towns, has in many cases considerably changed, with many becoming commuter towns, places to retire, or tourist destinations (Skerratt et al., 2012). As a consequence social connections that were traditionally created through a sense of shared identity no longer provide the social capital that binds these communities together.

Rural economic and social challenges might also offer opportunities. Although geographical distance creates an obstacle to many economic or social activities (Pateman, 2011), community cohesion deriving from a historical commitment to self-help born from necessity has led to high levels of trust and active civic participation within rural communities (Skerratt et al., 2012). Rural communities are often characterised by strong social networks, embeddedness and social movements (Jack and Anderson, 2002) as well as possessing strong mutual knowledge, sense of community and social cohesion (Dale and Onyx, 2005, Woods, 2003, Woods, 2008). Rather than competing, many rural businesses embrace the concept of co-opetition (i.e. collaboration between business competitors in the hope of mutually beneficial results), and draw upon rural strengths to specialise particularly within niche markets (Steiner and Cleary, 2014). Rural locations are often characterised by higher business density per head of population than urban areas (Steiner and Atterton, 2014), although it is not clear whether these high levels of entrepreneurship derive from push or pull factors (Kirkwood, 2009). Nonetheless, these features could potentially be harnessed by rural social enterprises to address economic challenges, issues associated with diminishing public services and weakening social connections and, as such, support rural development.

### 2.2 Social enterprise domain

Social enterprises are businesses that trade for a social purpose. Their primary objectives are social and the profit or surplus generated by business activities is used to these objectives (Chell, 2007). Many commentators identify two key characteristics of social enterprises including *enterprise orientation* (Dees, 1998) and *social and/or environmental aims* (Borzaga and Defourny, 2001). Although not common to all the academic literature, particularly in the United States, a third characteristic identified by many scholars is *social ownership.* Social enterprises are independent, run by communities or individuals and not governed by the state, with profits being distributed for societal benefit (Nyssens, 2007). Beneficiaries of social enterprises tend to be specific community groups but the outcomes of their activities frequently bring broader benefits to local geographical communities (Defourny and Nyssens, 2010). It is helpful to see social enterprises as an umbrella term capturing a wide range of organisational types, operating in different areas of the economy. Some operate in private markets similar to other businesses (Steiner and Teasdale, 2016). Some deliver public services under contractual arrangement (Teasdale, 2012a). Some are more akin to voluntary organisations and mutual societies, and may rely more on volunteer labour and grant funding in recognition of their role in developing social cohesion (Munoz et al., 2015). Others provide employment for disadvantaged groups, and may require subsidies from government to achieve this (Teasdale, 2012b). Many social enterprises combine several objectives, and rely on a diverse range of funding to achieve their social mission.

This ‘hybrid’ nature becomes more understandable when seen from the perspective of a rural social enterprise seeking to develop the local community, rather than necessarily being created to address a particular social problem. A hypothetical example could be a community group that owns a village hall and holds meetings to discuss local challenges. The group recognises a need for rural transport so that villagers can attend local markets and the hospital in the nearby town. The community group develops a bus service that, in time, receives a contract from the local council. At the same time, the community wants to enhance employment opportunities for young people to avoid their outmigration, and does this through the community transport scheme by creating links with local market towns and creating job vacancies as bus drivers. To utilise available resources, the transport scheme expands to provide delivery to a local community village shop which, together with community support and free labour, is able to break even and provide services locally.

It would, therefore, seem that social enterprises may offer a holistic approach to tackling interconnected problems in rural communities. They are culturally embedded and benefit from adaptive capacity (Steinerowski and Steinerowska-Streb, 2012). Valchovska and Watts (2016) indicate that the community presence in enterprise creation and development, aspects of social structure, social capital and cultural values all impact on the social enterprise activities. The cooperative ownership of community enterprises creates economic opportunities otherwise not available in local settings (Sodhi and Tang, 2011). The success of social enterprises depends upon dedicated groups of people and their collective action (Pickernell et al., 2007, Williams, 2007). Set against this however, there may be a very small pool of people in rural communities who are able and willing to get involved (Munoz et al., 2015). Munoz and Steinerowski (2012) also highlight that the necessary skills may be lacking in rural communities, particularly with regards to more business-focused social enterprises.

Social enterprises currently occupy a niche place in rural communities (Farmer et al., 2008). Despite the rhetoric emanating from within the social enterprise sector and governments, most social enterprises are unlikely to ever be sustainable solely from selling goods and services (Clark et al., 2007). In part this might be attributable to a traditional hostility towards more business-focused approaches, particularly among the more voluntary sector-type organisations and people where there is often resistance to risk and/or capitalism (Whitelaw, 2012). Sustaining social enterprises in rural locations is therefore complex and requires significant amounts of nurturing and support (Whitelaw, 2012). Those running social enterprises need to understand and adapt not just to local needs but also the extra-local, vertical context incorporating the policy, legislative and sectoral landscape (Skerratt, 2013). This landscape is not always conducive towards social enterprises. Thus, if we want to use social enterprises as a vehicle for rural development, we may also need to consider how to adapt this policy, legislative and sectoral landscape (Smith and McColl, 2016) towards an approach that is people-centred and where people co-design and co-produce locally tailored services rather than trying to substitute for unsuccessful state or local authority provision (Zografos, 2007).

### 2.3 Policy domain

Scotland is one country that, on the surface at least, offers a policy environment that is favourable for social enterprise and is conducive towards a cohesive approach to tackling social and economic issues. The Economic Strategy for Scotland (2015) emphasises that increasing growth and tackling inequality are mutually supportive, and that reducing inequality is vital to deliver sustainable economic growth over the long term (Scottish Government, 2015b). This commitment to ‘inclusive growth’ and addressing inequality is central to the wider policy agenda, including public service reform based on co-production and focusing on preventative interventions (Scottish Government, 2011); a focus on regenerating local democracy; emphasis on ‘bottom-up’, and ‘place-based’ and ‘asset-based’ approaches to local development, for example, through the Community Empowerment (Scotland) Act (2015) (Scottish Governmant, 2015a) and the Land Reform (Scotland) Act (2016). The Procurement Reform (Scotland) Act 2014 is also expected to give advantages to social enterprises in bidding for public contracts, by strengthening the extent of community benefit clauses on environmental, community and social grounds. It is argued that through identifying locally based solutions and engaging communities and co-producing tailored services to those in need, social enterprises can contribute to achieving National Outcomes (Scottish Government, 2007). To facilitate this, Scotland has a ten-year social enterprise strategy (Scottish Government, 2016a) to make Scotland an environment where social enterprise can play a central role in the inclusive growth strategy. However, none of these policies are designed to support specifically rural social enterprises and ‘rural proofing’ of existing and new policies may not be sufficient.

Flourishing support structures for Scottish social enterprise include more than twenty national Social Enterprise Networks and support organisations. This infrastructure combined with the favourable policy environment would seem conducive to developing social enterprise. The social enterprise census for Scotland suggests a burgeoning sector with 5600 social enterprises in Scotland generating an annual income of £3.8bn, creating more than 80,000 full-time equivalent jobs and engaging thousands of volunteers. The census also shows that social enterprises are overrepresented in rural Scotland. Although just 18% of Scotland's population live in rural areas, they are home to one-third of all Scottish social enterprises. Therefore, Scotland would seem an ideal arena whereby the potential for rural social enterprise might be realised.

**Fig. 1:**
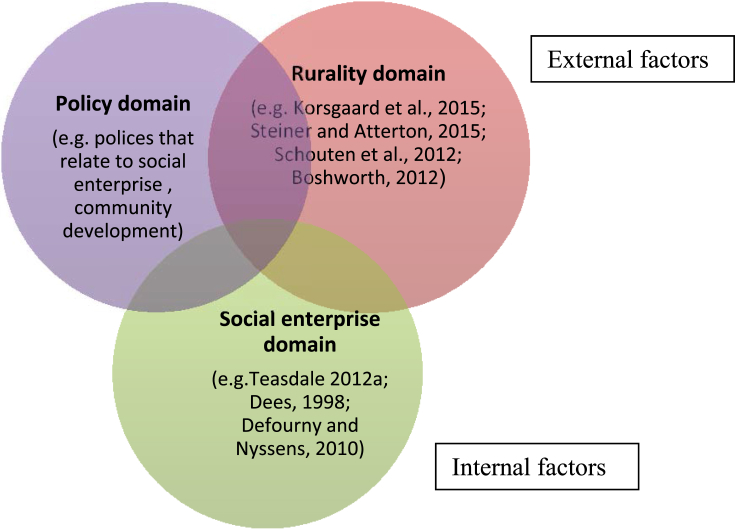
Key factors influencing development of rural social enterprise.

From this review of three overlapping bodies of literature we see that rural social enterprise development can be considered as influenced by three domains. *The geographic (or rural) domain* highlights that rural areas face particular challenges but may also offer particular opportunities by virtue of (comparatively) high levels of entrepreneurialism, co-operation, civic participation and dense social networks. *The social enterprise domain* highlights that rural areas appear well-suited to the development of social enterprise as they potentially offer a holistic approach to tackling interconnected problems in rural communities through drawing on the local resources prevalent in rural areas. In many countries, and particularly in Scotland – the site of our research – *the policy domain* would appear favourable to the development of social enterprise. These three domains are presented in Fig. 1 and are used as a conceptual framework helping to understand how to unlock potential of rural social enterprise. There is extensive literature covering each of the domains separately as well as relatively substantial literatures covering two domains – for example rural policy, or social enterprise policy. A smaller set of literature discussed previously relates to the intersection between rurality and social enterprise. The key to unlocking the potential of rural social enterprise would seem to lie (conceptually speaking) at the intersection between these three domains. To the best of our knowledge, ours is the first paper focusing on the intersection on this area. It is helpful to conceive of the rural and policy domains primarily (but not exclusively) as external environments structurally influencing the development of social enterprise, whether through policies, or economic conditions, and operating mainly outside of the control of the individual social enterprise. Conversely the social enterprise domain can be seen as representing those areas where there is still space for individual social enterprise agency. Here social entrepreneurs are able to, for example, choose to support particular client groups, select appropriate legal forms and governance structures, and address the balance between social and commercial goals. Of course it is important to recognise that these three domains are interconnected. The legal forms and governance structures available to the social enterprise are a consequence of decisions taken in the policy domain. The selection of particular client groups in need of support is influenced by rural economic and social conditions. This conceptual framework served as a starting point for our exploratory research aiming to better understand the potential impact of social enterprises on rural development, the opportunities and challenges they face, and to consider how local experts think the potential for rural social enterprise might better be realised.

## 3 Methodology

### 3.1 Study context

**Fig. 2:**
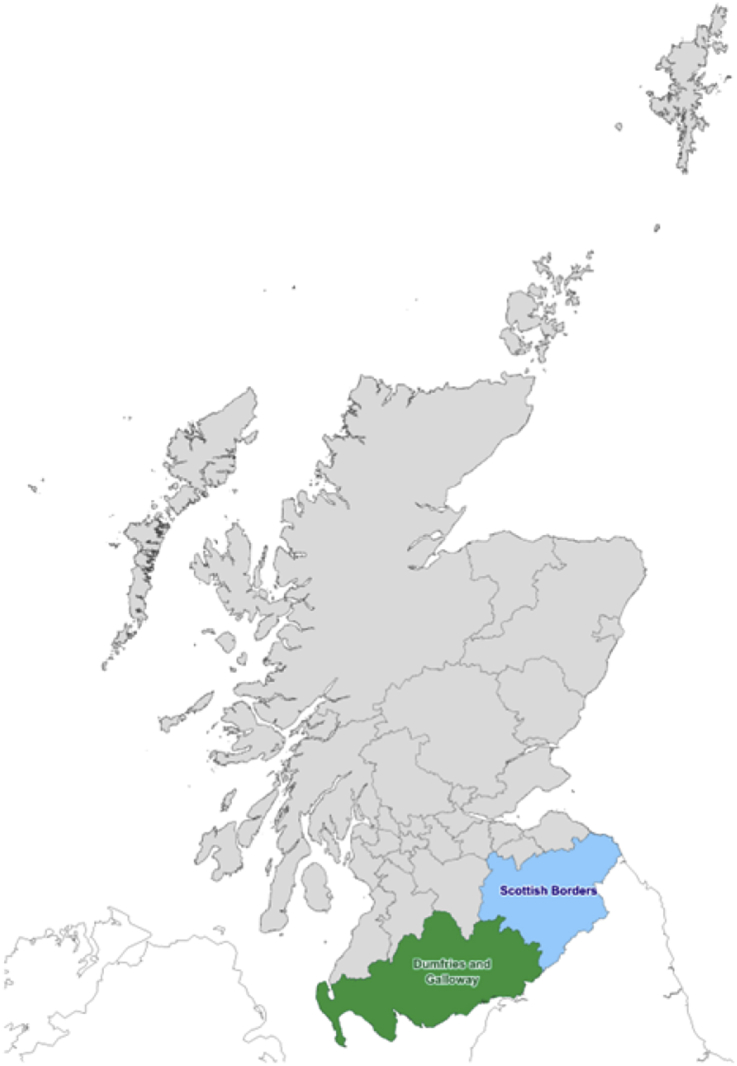
Map of Scotland and data collection areas including Dumfries & Galloway and Scottish Borders. **Source:** A map developed for the purpose of the study. **Copyright:** Geographic data reproduced under the terms of the UK Open Government License.

To develop our conceptual framework, we conducted an exploratory study aimed at developing an understanding about rural social enterprises, how to unlock their potential and to identify future research directions in the field. As such, the study did not intend to be generalizable in its scope but focused on exploring our framework in a rural context. Our research took place in the rural South of Scotland and its two local authorities of Dumfries & Galloway and Scottish Borders (Fig. 2). The study context was selected due to interesting economic and geographical features of the region. The Scottish Index of Multiple Deprivation reveals that the regions have a high proportion of deprived areas. Addressing social and economic challenges through traditional public service delivery is often too expensive due to the rurality of the regions and high per capita costs. The regions face the ‘typical’ rural problems presented in the opening section of this paper. Set against this, although just 5% of the Scottish population live in the South of Scotland, 8% of Scottish social enterprises are located in the region (Social Enterprise Census 2017, 2016). This suggests that the area represents a fertile ground for social enterprises and/or that they have a high level of unsatisfied needs that social enterprises try to address.

### 3.2 Research methods

We conducted conversional-style interviews (Lavrakas, 2008) with social enterprise stakeholders including local social entrepreneurs, managers and employees, members of social enterprise support organisations, those involved in social enterprise networks and regional politicians responsible for influencing social enterprise strategies and policies. A list of potential interviewees was provided by a regional social enterprise support network. They were identified with two criteria in mind: (i) their work had to be located within the study area, and (ii) they had to be directly affected by and/or involved in influencing social enterprise environment. This ensured that the interviewees were familiar with the local social enterprise context. Respondents were guaranteed anonymity to allow them to speak more freely and in total 11 interviews were conducted. A topic guide for data collection and analysis was developed based on a review of policy and academic literature. Examples of the questions are presented in Table 1.

**Table 1:** Example of interviewee questions.

Enquiry Topic	Example of Questions
Impact of rural social enterprise on rural development	• Please explain the importance and role of social enterprises in the South of Scotland. • What is the contribution of social enterprise to social, economic and environmental development of the region?
Opportunities and challenges faced by rural social enterprises	• What are the key opportunities for social enterprise development in the South of Scotland? • What are the key challenges and threats to social enterprise development in the South of Scotland?
Possible development areas	• What needs to be done and by whom to address the challenges and make the most of the opportunities?

Interviews lasted up to one hour and were recorded, with consent, using a digital voice recorder. In addition, key relevant notes were taken during the interviews. All data were coded, categorised and analysed manually using the constant comparative method approach (Glaser and Stauss, 1967). This involved listening to the recorded interviews to identify key themes of a discussion.

## 4 Understanding rural social enterprise

Our interviewees described the perceived impact of rural social enterprises on rural development, identified opportunities for the development of rural social enterprises and highlighted a number of challenges facing the enterprises. These three areas help to inform our discussion around unlocking the potential of rural social enterprise and the future rural social enterprise, and to draw conclusions.

### 4.1 Impact of social enterprises on rural development

#### 4.1.1 Economic resilience

Job creation was indicated as particularly important for rural areas where employment opportunities are limited, dependence on public sector jobs is high and where business opportunities are perceived as scattered: *‘twenty jobs were created because of this [social enterprise community] buy out. This is really important as its a very remote area and there are no jobs locally. So it gives opportunities for people to work locally and not to travel long distances'* (I5). It was suggested that rural social enterprises can bring significant investments to local areas. By selling local produce and generating employment, social enterprises help to keep money in the rural setting. Interviewees also indicated that many social enterprises fulfil very specific needs in communities providing local services that otherwise would not be provided. These functions could theoretically also be provided by commercial rural businesses (Steiner and Atterton, 2015) but social enterprises might also play an important role in addressing rural market failure, due in part on their ability to draw upon a wider resource mix from cohesive local communities.

#### 4.1.2 Community cohesion

Interviewees highlighted that rural social enterprises can both draw upon, and stimulate, voluntary and collaborative community culture, thus binding communities together. They may provide support to vulnerable groups of people using an entrepreneurial approach and create opportunities for people, including volunteering, that make a contribution to social cohesion of rural locations. Partially, this community cohesion derives from the way that social enterprises function: they are run by local people and for local people, and are often able to operate at a low cost through utilising voluntary commitment from local residents to maintain services. Consequently, common purpose becomes a key motivation for joined action and local integration: *‘social enterprises* support *vulnerable groups of people using entrepreneurial ways, creating opportunities for people. Volunteering opportunities make a massive contribution to social cohesion of the community, services for young people making the community more attractive for younger but also more attractive place to live for those with children who consider moving here. There is this intergenerational aspect of it where people have a chance to meet and work together. This relates to social sustainability’* (I6). This community cohesion aspect is frequently associated with voluntary organisations (Milligan and Fyfe, 2005). Rural social enterprises may represent an alternative approach also embedded in local communities that aims to tackle economic as well as social issues.

#### 4.1.3 Added value

Interviewees emphasised that the importance of social enterprises can be expressed through the long-term effect the organisations have on their local community. For instance, through providing support to and building skills amongst young people, social enterprises ensure that the group gains a practical experience that can influence their future economic life as well as the life of a community to which they contribute: ‘[this social enterprise] *collects and recycles books, reducing land fill and carbon emissions, provides training and employment to people who otherwise would be using benefits and some health services. So they are reducing the burden of employability problems. So there is a social added value. And there's a number of organisations like that who have social impact’* (I4). Arguably, commercial businesses, public sector and voluntary organisations also generate added value but social enterprises do that in a more integrated way with each social enterprise being involved in business and voluntary activities at the same time while frequently filling gaps of services previously delivered by the public sector. The added value also comes from this interconnectedness between different needs and operational aspects. Social enterprises do not operate in terms of policy (or academic) silos but, especially in the rural context, attempt to flexibly address local challenges. The nature of rurality means that small interventions can have a significant impact on a community: *‘the* [social enterprise] *generates economic income. In relation to environmental sustainability, it provides essentially a recycling service where people can donate unwanted goods that are then affordable for others. So there is a multiple effect, ripple effect. It seems like a small scale amateurish activity but it does make an impact in a small environment’* (I6).

### 4.2 Opportunities for rural social enterprise development

#### 4.2.1 Adapting to policy environment

All interviewees saw a key role of social enterprises being in the delivery of rural services such as health and social care, in raising employability, as well as in providing support to other public service organisations to help in designing innovative customer-friendly services. It was acknowledged that social enterprises could provide a number of preventative services as well as training and employment opportunities offered to those disadvantaged in society, thus potentially reducing future public spending, improving the health of participants, and reducing the burden on public services: *‘in the current economic climate we have to explore other ways of service delivery alternative to public sector delivery. Social enterprises have to be key to that’* (I7). Some interviewees talked about the how the Procurement Reform (Scotland) Act (Scottish Government, 2014), which involves taking into consideration the wider community benefits associated with public contracts, would create opportunities for rural social enterprise to deliver services. Other policy changes in health care such as Health and Social Care Integration (Scottish Government, 2016b) and Reshaping Care for Older People (Scottish Government, 2013) were highlighted as providing new opportunities for rural social enterprises. The Scottish policy environment was therefore seen as conducive to rural social enterprise. While these policies are specific to Scotland, it is notable that they can be seen as part of a broader global movement towards reforming commissioning and procurement arrangements to make governance more conducive towards co-production (Pestoff et al., 2013).

However, interviewees expressed demand for a more tailored support structure that understands local rural characteristics, and is familiar with the social, economic and environmental challenges and opportunities seen in rural communities. One need identified by the interviewees referred to creating regional incubation spaces: *‘if public sector partners are serious about developing social enterprises, we need to invest in incubation of ideas. Incubation is not new, it doesn't need to be a place, it can be virtual, preferably both – including a physical place with workshop and facilities* supporting *start-ups, with mentors and places where people can meet’* (I7). These kind of social enterprise hubs do exist in urban locations (Steiner and Teasdale, 2016), but are frequently inaccessible to rural communities.

#### 4.2.2 Awareness of local problems

As social enterprises arguably tackle local challenges utilising local resources, it was suggested they could provide more tailored and efficient services that would fit the rural context better than top-down initiatives. For instance, many areas in rural Scotland have a high proportion of older people. Due to the growing needs of the ageing population, health and care services represent an area in which social enterprises could expand. It was stressed that some retirees want to remain active and develop community enterprises: *‘there are people who retire with business background and they want to make a difference to their community, they want to a make social contribution and commitment to social development’* (I6). Hence, there are opportunities to use the skills and knowledge of older people to facilitate the development of socially entrepreneurial community projects (Farmer et al., 2011).

#### 4.2.3 Using the advantages of the rural context

Many participants talked about creating rural-based social enterprises involved in forestry or food related activities such as farming, agriculture or food processing – all industries traditionally associated with rural locations. The social enterprises could, for example, produce eco-friendly quality food, promote a healthy diet, and distribute food locally, reducing environmental footprint, thus stimulating wellbeing. One participant referred to farms that operate as social enterprises integrating disadvantaged people into the workforce and helping to reduce their social isolation. Interviewees also suggested that rural social enterprises can support the natural environment through looking after local land and adequately protecting its biodiversity. They might also create rural hubs for the region selling local produce. Finally, interviewees referred to better utilisation of underused existing resources such as village halls or old village shops that represent opportunities for social enterprises helping to restore rural life. Rural social enterprises were presented as organisations that creatively utilise available resources and local knowledge, and so can turn rural shortcomings into opportunities: *‘the rural geography of the region creates remoteness from the main services that are provided in the larger more urban areas. Therefore communities look to provide them for themselves, this provides a lot of social benefits for a community’* (I1).

#### 4.2.4 Partnership collaborations and enterprising development strategies

Interviewees suggested that the benefits and impacts of rural social enterprises can be enhanced by working together on joint projects with public, private and other third sector organisations: *‘partnership between organisations is an opportunity to generate income though contracts that sole enterprise would not be able to deliver’* (I9). It was suggested that replacing competition with partnership in order to be able to deliver contracts might bring mutual benefits. It remains to be seen whether the apparent move in Scotland towards procurement arrangements more conducive to co-production might facilitate this. Such collaboration need not occur only between organisations engaged in public service delivery. It is widely recognised in the rural business literature that collaboration between businesses can make them and their industries more sustainable (Steiner and Cleary, 2014). Participants noted that currently there is not enough cross-sectoral collaboration and that social enterprises should be better at sharing expertise and best practices, and learning from both success stories and failures.

Some participants referred to a need for those running social enterprises to better understand specific markets, competitors, efficient management and leadership. In order to become more ‘business-like’, those responsible for developing and running rural social enterprises might require training in the area of finance, marketing, selling and tender writing as well as peer support and study visits. According to some interviewees, those in charge of rural social enterprises need to focus on developing a long-term strategy, better management of finance issues, becoming more enterprising, delivering and charging for services appropriately, being more confident and understanding the value of their work. Interviewees also suggested that rural social enterprises leaders should be more ambitious, adopt growth strategies and go beyond their immediate geographical boundaries. For instance, rural social enterprises could and should take advantage of urban markets to overcome challenges of low population rural customer base.

### 4.3 Challenges facing social enterprises

In addition to the highlighted opportunities, social enterprises in our study area faced a number of challenges that might hinder their development and their potential impact on rural development.

#### 4.3.1 Rurality as challenging geographic context

The majority of interviewees echoed the academic literature in stressing that the remote and rural context creates specific barriers including challenges relating to low population density, isolated communities, lack of larger town centres, long distances to travel, a lack of efficient public transport and well-developed infrastructure. Those running social enterprises highlighted that income generation in rural areas is difficult. They suggested that to be successful, social enterprises need to be well-placed, well connected to local communities and develop their reputation over time. Interviewees said that the development of social enterprises requires long-term commitment and well-planned investments that consider geographical limitations. They also indicated that support networks are not easily accessible for remote and rural social enterprises and that the organisations find it challenging to communicate information with ‘the right people’. Networking and face-to-face knowledge dissemination is often problematic. Interviewees highlighted that there are very few large social enterprises in rural locations, and that small social enterprises face challenges in acquiring public contracts and, thus, difficulties in attaining ongoing access to income and reaching financial sustainability. However, the small scale of rural social enterprise might be appropriate for the local context: *‘There is a growth potential of new small-scale niche social enterprises in the whole range of communities across the regions. It's part of the wider agenda for rural sustainability, financial, social and environmental sustainability, economic and population. People in rural areas are trying to solve slightly different problems than people in urban areas and social enterprises can help achieving good results'* (I9). It would seem necessary to move away from ‘scaling up’ as a goal, and instead to consider how policy can support more small (and cohesive) social enterprises that collectively make an important contribution: *‘some social enterprises might be small and generate only a few jobs but these few jobs are important in the rural economy’* (I7).

#### 4.3.2 Policy rhetoric vs. reality

Participants claimed that commitment from the Scottish Government and favourable national policies supporting social enterprise development often does not translate into local support: *‘*support *for social enterprises at national level is extremely good. We've got a supportive environment nationally. But it is often not well translated into a regional level. Local authorities and procurers and commissioners might be less helpful. There is commitment from the Scottish Government but it often doesn't transfer into the local level’* (I9). Other studies have highlighted this gap between rhetoric and political surrounding the Scottish approach (Cairney et al., 2016, Mazzei and Roy, 2017).

Interviewees stressed the Procurement Reform (Scotland) Act 2014 does not require local authorities to consider breaking up contracts into smaller sub-contracts. A large proportion of public contracts cover entire local authority areas and their size and value preclude rural social enterprises (that are predominantly small) from tendering. Many rural social enterprises have no experience in bidding for contracts from public agencies creating know-how entry barriers. Participants argued that public contracts should be designed in a way to enable smaller local social enterprises to access them and, as such, produce locally responsive services.

#### 4.3.3 Not enough enterprise?

All participants indicated that social enterprises have competing social and commercial objectives which make management difficult: *‘it is a difficult business model to get right because it is necessary to compete between commercial viability and targeting social challenges’* (I8). These challenges are particularly evident in rural locations where a small customer base often limits opportunities for income generation. Many social enterprises receive grants to support their activities. Interviewees expressed their concerns that in the future there will likely be less grant support and, therefore, social enterprises must generate more commercial revenue: *‘social enterprises should be less* grant *dependent … They shouldn't start from an assumption that they will be* grant *dependent.* Grants *shouldn't be the main source of income. It should be an addition’* (I4). However, it is important to recognise that social enterprises deliver highly diversified services, some of which are less commercially viable (but perhaps more socially necessary).

Many interviewees stressed that often it is not clear who is responsible for social enterprise development, who takes the financial risk associated with running a social enterprise and who owns the organisation: *‘ownership might be problematic e.g. if it is successful – who owns it; or if it fails – who is responsible for that’* (I8). According to participants, social enterprises managed by rural communities are frequently not commercially focused, and their decision-making process may be slow and inefficient. These create obstacles for social enterprises to become viable social businesses. The majority of interviewees emphasised that in terms of truly enterprising rural social enterprises, the sector is underdeveloped and that rural areas need more entrepreneurial individuals with social vision. Interviewees claimed that finding experienced and well-skilled employees to work with and support social enterprise development in rural settings is frequently challenging and that it is essential to build entrepreneurial communities that are actively involved in designing entrepreneurial solutions addressing existing local challenges: *‘it is necessary to find the champions, leaders, entrepreneurs – nurtured in the community – and the community should* support *them’* (I5).

Finally, it was suggested that there is a cultural aversion to risk across rural social enterprises, particularly a reluctance to replace grants with loans: *‘social enterprises can't be entirely commercial as they can't make purely commercial decisions. Frequently they are managed by a board of directors who are not very commercially focused. Making decisions is impossible within a community group. There's always a conflict because community councils tend to be conservative and don't want to take risk, they want get a lot of people involved in decision making so the process is very slow. If you're a sole trader you make a quick decision but in social enterprises you can't do that’* (I8). Many rural social enterprises may lack an entrepreneurial approach and/or they fear experimentation and innovations. Although different levels of risk might be appropriate in different situations and contexts, risk aversion and resistance to change might leave rural social enterprises in a position where the organisations serve only niche markets or fail to address changing problems.

#### 4.3.4 Understanding the diversity of social enterprise funding for social enterprise development

Interviewees had different opinions about reliance on grant support. Some indicated that grants might create a ‘dependency culture’ slowing down innovation and limiting organisational long-term sustainability: *‘Many social enterprises die because of lack of* funding support. Grant *dependency is not good though. Old model of* supporting *communities needs to be replaced with a more enterprising new model adapted to the current times’* (I2). Others suggested that grant funding for rural social enterprises is essential and it should include, for example, early-phase start-up awards as well as follow-on financial support options. Replacing grants with interest free or low interest repayable loans was suggested by some interviewees, as this might encourage rural social enterprises to be more business-like and resourceful. Participants said that *‘there are* funding *opportunities now and again but they are not consistent’* (I6) and, therefore, it is difficult for social enterprises to rely on them. Access to funds in remote areas is difficult and frequently, according to interviewees, there is lack of understanding of rural issues amongst funding organisations. It appears that different types of social enterprises get funding in different ways, and that the funding environment needs to be tailored towards the diversity of social enterprise.

#### 4.3.5 Danger of over-reliance on business approaches

Many participants claimed that the push towards a ‘business-like’ environment creates competition between social enterprises and that there is a risk of replacing social purpose with financial targets: *‘there is also the current threat of voluntary organisations retaining their social values when they become social enterprises. The new ‘business-like’ environment creates an idea of competition and that the sharing of information and resources that took place as a voluntary organisation no longer happens as a social enterprise. The presence of business-minded people around the table can threaten the social purpose’* (I1). Interviewees said that many third sector organisations are forced to move away from grant funding to turn into social enterprises. While some participants suggested that this ‘competition’ between social enterprises and voluntary organisations might increase innovation and encourage entrepreneurship and resourcefulness of rural social enterprises, others claimed that that this business-like approach might not work well in rural communities due to the embedded informal help culture. One participant stressed that turning everything into ‘business’ might bring negative long-term effects on the community development. Again the conflicting perspectives hint at a need to recognise and respect the diversity within social enterprises, and also to recognise the symbiotic relationship between social enterprises and the wider third sector in tackling social issues, rather than seeing them as substitutes.

## 5 Discussion

Through reviewing existing policy documents and academic literature, and drawing on primary data from rural Scotland, this paper summarises the challenges to, and opportunities for, rural social enterprise development, and thematically aligns them with the rural, social enterprise and policy domains identified as part of our conceptual framework (Table 2) This enables us to identify gaps in our research knowledge and suggest pathways to unlock the potential of rural social enterprise.

**Table 2:** Challenges and opportunities to rural social enterprise development associated with the rural, social enterprise and policy domains.

Factors influencing rural social enterprise	Challenges to rural social enterprise development	Opportunities to rural social enterprise development	What needs to happen to unlock potential of rural social enterprise?	Research gaps
Rural domain	- Rurality as challenging geographic context	- Using the advantages of the rural context	- Turning challenges into business opportunities	- Explore how rural industries and local resources could create social value and address local challenges - Examine successful examples of turning underused rural resources into socially entrepreneurial ventures
Social enterprise domain	- Not enough enterprise? - Danger of over-reliance on business approaches	- Awareness of local problems - Partnership collaborations and enterprising development strategies	- Efficient use of local resources to address local problems - Incorporation of enterprising business approach while remaining small, informal and flexible	- Explore strengths and weaknesses of different forms of social enterprises that exist in the rural context, and how these forms utilise and adapt to local settings - Test a variety forms of funding and grants targeted at social enterprises addressing different local service gaps
Policy domain	- Policy rhetoric vs. reality - Understanding the diversity of social enterprise funding for social enterprise development	- Adapting to policy environment	- Recognition in policy documents that rural social enterprise development requires relevant strategies that fit with the nature of rurality - Need for social enterprise to be responsive towards policy directions	- Explore how different policy contexts create different rural social enterprise development outcomes - Explore if and how rural social enterprises proactively use policies to further develop their activities

Firstly, with respect to the ***rural domain***, our interviewees indicated that with appropriate guidance and support structures, many rural challenges and needs could be transformed into opportunities for social enterprise development. For instance, the ageing population could act as a promoter for developing social enterprises in health and care service provision. Regions characterised by a high degree of rurality could take advantage of rural settings and develop social enterprises involved in rural industries such as food production initiatives, tourism or renewable energy projects. Aspects of turning needs into opportunities should be researched and, if proven, promoted. The challenge lies in supporting rural communities to recognise and address their own needs through social enterprise, rather than dictating, through the policy and funding environment, which needs should be addressed.

With regard to research, potential areas of interest associated with the rural domain concern how rural industries and local resources might be used to create social value and local challenges. One way of doing this might be to focus on successful examples where underused rural resources have been transformed in socially entrepreneurial ventures. This requires a greater contextual understanding of the rural environment than is apparent in some early social enterprise studies that focused on the hero entrepreneur narrative (Nicholls, 2010). In essence rurality needs to be treated, at least in part, as an explanatory variable.

Secondly, looking at the s***ocial enterprise domain*** and characteristics of rural social enterprise, many participants in our study spoke of the dangers of ‘grant dependency’ and ‘risk aversion’. There are dangers that these might leave rural social enterprises in a position where the organisations serve only niche markets or operating in markets in a state of permanent precarity. Others, however, warned of the dangers of becoming too commercially focused. Some social enterprises, particularly those operating in relatively profitable industries may not require long-term grant support. But others operate in areas which may never be commercially viable without outside support – for example providing rural transport in remote areas. Recognising and celebrating the diversity of the social enterprise landscape requires also accepting the diversity of the funding mechanisms necessary to support different types of social enterprise; providing business support for ideas with commercial potential, opening up access to public contracts for smaller social enterprises, but also providing grant funding to those organisations delivering essential rural services at a loss.

Collectively, it would seem that rural social enterprises could help communities to take control of and tackle complex social, economic and environmental challenges. Similar to those located in urban locations, rural social enterprises might co-operate, pool and share resources, buy and sell from each other and jointly bid for contracts. This would help to unlock resources, provide scale where needed, minimise costs, and stimulate innovation, while keeping money circulating within social enterprises. Drawing on available resources, rural social enterprises could potentially create locally responsive services that fit the rural context better than top-down initiatives. Being ‘small’ need not preclude social enterprises from getting larger contracts. Through partnerships with other organisations and working in larger consortia, social enterprises could tender for bigger public contracts. At the same time, public contracts could be designed in a way to enable smaller local social enterprises to access them and produce locally responsive services. This represents a challenge to public service procurement as dealing with a large number of small contractors is challenging. Hence, we observe tensions between ‘what is good’ for rural development and what is practical (or easier) in terms of implementing procurement processes.

In order to unlock their potential, interviewees suggested that rural social enterprises need a level of flexibility in integrating formal business activities with the informal action of rural residents. It therefore seems that the ‘traditional’ approach to ‘scaling up’ and achieving economies of scale might not apply to rural social enterprises. Instead, as Bovaird (2014) highlights, collaborations between social enterprises and between networks of social enterprises and public sector providers can lead to economies of scope, particularly where strong trust-based relations within communities can harness self-help and the co-production of services. This process is facilitated when competition between organisations, particularly for financial resources, is reduced.

As regards future research connected to this domain, we need to better understand the diversity of social enterprises in the rural context, how this diversity might relate to the policy and rural domains, and the strengths and weaknesses of different forms of social enterprise. This can lead to policy-orientated research aimed at assessing the suitability of different funding mechanisms for different types of social enterprises aimed at achieving different outcomes. Here it is necessary to treat social enterprise as operating within an ecosystem which can be supported through policy measures, while recognising that policy initiatives in one area may have unintended consequences in other areas.

Finally, and in relation to the ***policy domain***, due to specific *modus operandi* and the rural context in which they are embedded, rural social enterprise may enhance economic resilience and social cohesion. Although private, public and voluntary organisations also generate value, rural social enterprises may potentially do this in a more integrated way through being involved in business and voluntary activities at the same time, across a wide range of social and economic issues. This ‘big picture’ approach to tackling local issues at the local level through single (or groups of) social enterprises permits flexible approaches addressing local challenges, and recognises the interconnectedness between different rural needs and operational aspects. Interviewees suggested that social enterprise can benefit rural communities through being able to operate across a wide range of policy silos. To provide efficient support to this process, public bodies would need to better understand the wider benefits generated by individual rural social enterprises, but also to recognise their internal and external diversity. Simultaneously, rural social enterprises might aim to become more reactive towards and take advantage of new policy initiatives that support social enterprises.

Research emanating from this domain might fruitfully begin to understand how different policy contexts create different social enterprise ecosystems. Comparative research erring towards natural experiments offers considerable potential here, although it is important to recognise that no two rural domains or social enterprise domains are identical. Nonetheless the development of local social enterprise strategies and/or policy approaches in much of Scotland highlight the exciting opportunities for contextually aware research aimed at understanding the impact of policy. Other studies might seek to explore how social enterprises use policies and adapt to policy landscapes to further their own development (see for example work in an urban context by Dey and Teasdale, 2016). Together these two approaches hint at the need to recognise the interdependence between policy environment and social enterprise, and to incorporate theoretical approaches that break down the structure agency dichotomy.

## 6 Conclusions

Although there is a significant body of academic literature on social enterprise, knowledge about *rural* social enterprise is largely scattered and undeveloped. The rural context matters and this has been acknowledged in academic literature for many years through publications in journals like *Journal of Rural Studies*. This does not mean that rural context is ‘good’ or ‘bad’. Instead, it shows that rurality brings specific opportunities and challenges that impact on how organisations like social enterprise operate. Understanding rural factors and their impact on social enterprises remains, however, limited. This paper marks an initial attempt to redress this gap and, based on the literature review, designs a conceptual framework for rural social enterprise development. We began to populate this framework using interviews with social enterprise stakeholders to suggest how the potential of rural social enterprises might be unlocked. This enabled us to identify gaps in knowledge which future research might fruitfully address.

Our conceptual framework identified that rural, policy and social enterprise domains need to be considered. Firstly, our paper alerts that the policy environment is extremely important in unlocking the potential of rural social enterprises. In order to create efficient policies supporting rural communities and rural social enterprise interventions, we need to better understand what we are dealing with. It seems that existing approaches to developing rural social enterprises are rarely evidence-based. This may have negative effects on rural social enterprise development. As public sector budgets face ongoing constraints, there is a pressing need to find more innovative and efficient approaches to local development. Decisions based on research evidence would help minimise risks of failure and increase opportunities for comprehensive rural community development that is receptive to both weaknesses and strengths of the rural context. Social enterprises, on the other hand, should be more responsive towards policy needs that shape directions of strategic national and regional aims and objectives as well as changes in the legislation that affect activities of social enterprises. It seems, therefore, that both policymakers and those running social enterprises need to collaborate better to become more efficient and bring mutual benefits. The impact of different policies on rural social enterprise development outcomes should be investigated to assess effectiveness of the policies in the rural context.

Our literature review suggested that social enterprise offers an approach to rural development that brings together elements of voluntary sector, public sector and mainstream business within a set of organisations displaying remarkable diversity but bound together by a commitment to social ownership. This is consistent towards policy moves in the direction of inclusive growth, and holistic approaches to tackling social problems. However, at the same time, it brings its own distinctive challenges to traditional ways of working, due to the need to understand the diversity of motivations, approaches, organisational forms and funding mechanisms within the diverse world of rural social enterprise, and adapting support structures and funding mechanisms appropriately to the organisation. Evidence presented by our interviewees suggests that at the local level, policymakers have not yet come to grips with the diversity of social enterprise, and how this very diversity may help tackle a diverse range of complex and interconnected social and economic problems.

Although financial sustainability is an issue faced by all social enterprises, our literature review highlighted that the rural domain imposes an additional commercial challenge on these organisations. From a policy perspective, a one-size fits all approach, whether based on grant funding, or encouraging social enterprises to become more commercially focused, is dangerous. Our data suggest that many respondents warn of grant dependency stifling innovation and risk. Equally however, our respondents also warned of the dangers of stifling traditional self-help activity built upon reciprocity and informality through overreliance on business-like approaches.

To avoid supporting only ‘privileged’ social enterprises that fit well with funders' understandings of what social enterprises are and do, it may be necessary to recognise that each social enterprise is different, in part a consequence of the particular rural context they operate within. A challenge for researchers and policymakers therefore is to understand how to support the different activities of social enterprises in different ways. This might require, for example, understanding of how grants might be used to support social enterprises delivering community cohesion, subsidies given to social enterprises employing disadvantaged workers, contracts given to social enterprises delivering public services, and business support and entrepreneurial skills offered to those social enterprises wanting to develop in private markets. Many rural social enterprises do some, or all, of these simultaneously. The ‘added value’ from this interconnected approach to tackling multiple rural issues simultaneously is what seemingly gives rural social enterprise the potential to play a truly transformative part in reshaping rural economy and society. But recognising and supporting this is particularly challenging for a public sector that has traditionally worked in silos to address specific problems, with economic development seen as separate to, for example, community transport, health and care service provision, or community cohesion (Cairney et al., 2016).

This paper has some limitations. Our empirical findings are based on a relatively small sample and the exploratory study data originates from two rural local authorities in Scotland. The empirical findings might be specific to the Scottish rural context. Although potentially limited in its generalizability, our paper does open up an agenda for future research in this under-researched field. As our conceptual framework derives from a wider international literature, it has wider application beyond the Scottish context. Future research might fruitfully seek to apply and refine this framework in other rural settings to gain greater understanding of contextual points of difference and similarity. When doing so, our paper highlights a need to move away from understanding social enterprise as a particular business model, and to better understand social enterprises as multi-scalar and multi-dimensional organisations that encompass multifaceted mechanisms for social, economic and environmental community development. This suggests different methodological approaches necessary to capture multidimensional aspects of rural social enterprises including rich qualitative studies such as ethnography as well as more traditional quantitative research methods aimed at generalisation. Relatedly, research should focus less on single social enterprises as self-contained systems, and pay more attention to their spatial and dynamic contexts.

## Appendix A Supplementary data

Data profile Data profile
